# Cardiorespiratory fitness and the metabolic syndrome: Roles of inflammation and abdominal obesity

**DOI:** 10.1371/journal.pone.0194991

**Published:** 2018-03-28

**Authors:** Anne-Sophie Wedell-Neergaard, Rikke Krogh-Madsen, Gitte Lindved Petersen, Åse Marie Hansen, Bente Klarlund Pedersen, Rikke Lund, Helle Bruunsgaard

**Affiliations:** 1 The Centre of Inflammation and Metabolism/ the Centre for Physical Activity Research, Rigshospitalet, University of Copenhagen, Copenhagen, Denmark; 2 Section of Social Medicine in Department of Public Health, University of Copenhagen, Copenhagen, Denmark; 3 Center for Healthy Ageing, University of Copenhagen, Copenhagen, Denmark; 4 National Research Centre for the Working Environment, Copenhagen, Denmark; 5 Department of Clinical Immunology, Rigshospitalet, University Hospital of Copenhagen, Copenhagen, Denmark; Universidad Pablo de Olavide, SPAIN

## Abstract

**Objective:**

Individuals with metabolic syndrome have increased risk of type 2 diabetes and cardiovascular disease. We aimed to test the hypothesis that a high level of cardiorespiratory fitness (CR-fitness), counteracts accumulation of visceral fat, decreases inflammation and lowers risk factors of the metabolic syndrome.

**Method:**

The study sample included 1,293 Danes (age 49–52 years) who from 2009 to 2011 participated in the Copenhagen Aging and Midlife Biobank, including a questionnaire, physical tests, and blood samples. Multiple linear regression models were performed with CR-fitness as exposure and plasma levels of cytokines and high sensitive C-reactive protein as outcomes and measures of abdominal obesity were added to test if they explained the potential association. Similarly, multiple linear regression models were performed with CR-fitness as exposure and factors of the metabolic syndrome as outcomes and the potential explanation by inflammatory biomarkers were tested. All models were adjusted for the effect of age, sex, smoking, alcohol consumption, socio-economic status, and acute inflammatory events within the preceding two weeks.

**Results:**

CR-fitness was inversely associated with high sensitive C-reactive protein, Interleukin (IL)-6, and IL-18, and directly associated with the anti-inflammatory cytokine IL-10, but not associated with tumor necrosis factor alpha, interferon gamma or IL-1β. Abdominal obesity could partly explain the significant associations. Moreover, CR-fitness was inversely associated with an overall metabolic syndrome score, as well as triglycerides, glycated haemoglobin A_1c_, systolic blood pressure, diastolic blood pressure and directly associated with high-density lipoprotein. Single inflammatory biomarkers and a combined inflammatory score partly explained these associations.

**Conclusion:**

Data suggest that CR-fitness has anti-inflammatory effects that are partly explained by a reduction in abdominal obesity and a decrease in the metabolic syndrome risk profile. The overall inflammatory load was mainly driven by high sensitive C-reactive protein and IL-6.

## Introduction

Physical inactivity has been identified as the fourth leading risk factor of mortality, causing 6% of all deaths globally [1]. Physical inactivity leads to accumulation of visceral fat [2] and is directly associated with systemic inflammation in cohort studies [3–5]. In accordance with this, regular physical activity has beneficial effects on factors in the metabolic syndrome [6,7] that reduces the risk of common chronic life style diseases such as cardiovascular diseases (CVD) [8] and type 2 diabetes mellitus (T2DM) [9]. Anti-inflammatory effects of physical activity are considered to play a role in protection of the metabolic health [10,11].

The metabolic syndrome is defined as a cluster of cardio-metabolic risk factors including elevated waist circumference (waist), elevated triglycerides, high blood pressure, insulin resistance and reduced high-density lipoprotein (HDL) [12]. Metabolic syndrome is accompanied by chronic systemic low-grade inflammation [13], characterized by a two- to fourfold elevation in circulating levels of inflammatory cytokines and acute-phase reactants [14]. Low-grade inflammation predicts the mortality risk in middle-aged and elderly populations [15]. Chronic inflammation is considered a central driver and a molecular link between components of the metabolic syndrome and CVD as pro-inflammatory pathways promote atherosclerosis [16–18]. Moreover, chronic inflammation links metabolic syndrome with T2DM as pro-inflammatory pathways induce insulin resistance [19]. Most epidemiological studies focus on C-Reactive Protein (CRP) and Interleukin (IL)-6 as biomarkers of chronic inflammation [20], however, IL-6 and CRP are likely surrogate biomarkers of tumor necrosis factor alpha (TNF-α) and IL-1 mediated activities [21]. Thus, TNF-α and IL-1β initiate inflammatory processes and constitute upstream triggers of IL-6 production, which again stimulates liver production of the acute phase reactant CRP [22,23]. A regulatory anti-inflammatory response (i.e. IL-10) inhibits the production or signalling of the pro-inflammatory cytokines [24,25]. IL-18 is a pro-inflammatory cytokine implicated in insulin resistance and the risk of T2DM [26]. IL-18 stimulates the production of interferon gamma (IFN-γ) [27,28], which in turn is likely to be implicated in the pathogenesis of atherosclerosis [29–31]. Both IL-18 and IFN-γ are cytokines that have been shown to be related to the metabolic syndrome [27].

Circulating levels of cytokines such as TNF-α and IL-6 are directly correlated with fat mass [32]. This relationship is considered to reflect spill over to the circulation of inflammatory mediators produced by adipocytes and cytokine producing immune cells, infiltrating the adipose tissue [14]. Visceral fat is more inflamed than subcutaneous fat [33] and the amount of visceral fat is more strongly correlated to the risk of developing CVD and T2DM than the amount of total body fat [34,35]. Measures of visceral fat mass is highly correlated with measures of abdominal obesity [36]. In accordance with this, measures of abdominal obesity such as waist, waist-to-hip ratio (WHR), waist-to-height ratio (WHtR) and truncal fat percentage (trunk fat%) are better risk markers than body mass index (BMI) [37,38].

CR-fitness is partly determined by genetic traits and partly by the amount of physical activity which can improve the maximal oxygen consumption and thereby increase the CR-fitness level [39,40]. We hypothesised that high levels of CR-fitness prevents accumulation of abdominal obesity and consequently inhibits the network of inflammatory pathways. We also hypothesised that this anti-inflammatory effect of CR-fitness would inhibit the development of the metabolic syndrome. The aim of this study was to test these hypotheses in a cohort of middle-aged Danes from the Copenhagen Aging and Midlife Biobank (CAMB) with an available detailed profile of inflammatory biomarkers including TNF, IL-1β, IL-18, IFN-γ, IL-6, hsCRP, and IL-10 [41]. Moreover, we investigated if an overall combined load of inflammation was more robust in statistical models, than single inflammatory biomarkers.

## Methods

### Study population and design

This study was based on data from CAMB, a cohort designed to study signs of early ageing in middle-aged Danes [41]. CAMB includes participants from three existing cohorts of which only one, The Copenhagen Perinatal Cohort (CPC), was used for the present study since it provides data on CR-fitness levels. CPC contains data from individuals born at the National University Hospital of Copenhagen between 1959 and 1961.

CPC included a total of 8,102 men and women in 2009–2011 at the CAMB follow-up. Of these, 5,196 individuals (age 49–53 years) were living in eligible geographical areas. A total of 1,718 individuals accepted the invitation and participated in the clinical examination, constituting a 33% participation rate in CAMB. For the present study, participants lacking CR-fitness data (n = 310) or blood samples (n = 90) or with BMI≤18.5 (n = 25) were excluded, leaving data on 1,293 participants (580 men and 713 women) for analysis.

The collection of data for CAMB included a comprehensive questionnaire, a health interview, a physical examination and blood sampling. The questionnaire collected data about health, use of medication, occupational social class and health behaviour. The physical examination was performed by educated staff following a standardized protocol that covered estimated CR-fitness, blood pressure, anthropometrics and a bioelectrical impedance analysis. Furthermore, non-fasting blood samples were collected [41]. The Ethical Committee of the Capital Region of Denmark approved the study (No: H-A-2008-126), and written, informed consent was obtained from all participants. CAMB is also registered at the Danish Data Protection Agency as a combined database (No: 2008-41-2938).

### Physical tests and metabolic syndrome

#### CR-fitness

To estimate oxygen consumption, a submaximal test was performed using an ergometer bike and CR-fitness level was calculated using the Aastrand nomogram [42]. The height of the seat was adjusted and participants were informed to keep a cadence of 60 pedal rounds per minute (Ergometer bike, Monark 939 E, Serial number: WBK 2740 20, Denmark, 2010). Heart rate was measured by a pulse belt (T31, Polar®, 2010) during the 16 minutes biking session. Subjects initiated the test by eight minutes light warm up on an ergometer cycle. Subsequently, the workload was increased in order to yield a stable pulse just exceeding 120 beats per minute, which was typically obtained after six minutes. When a steady state heart rate was achieved, pulse data was registered every half minute for two minutes, before the concluded. If a steady state was not achieved within six minutes the test was extended one minute or until steady state was reached. An average pulse was calculated from the four measurements made at the end of the session, and maximum oxygen consumption was estimated from the average pulse, workload and gender using the Aastrands nomogram [43].

Inclusion criteria covered all participants from CPC, who lived in eligible geographic areas and accepted the invitation as well as participated in the clinical examination.

Exclusion criteria included 1) moderately increased blood pressure (systolic >160mmHg and/or diastolic >100mmHg), 2) angina pectoris, 3) fever, 4) heart medication (beta blockers), 5) resting heart rate >120, and 6) having eaten a big meal, smoked cigarettes or performed hard physical work within the last hour before the test. After the test, data on CR-fitness were missing for a total of 310 participants, either due to the above listed exclusion criteria (n = 201) or to technical problems with the bikes (n = 109).

#### Body composition

Waist [44], WHtR [45], WHR [46] and bioelectrical impedance analysis of trunk fat% (SECA 201, Germany, 2009) (TANITA® MC 180, Japan, 2010) were used as measures of abdominal obesity to increase the robustness of our findings [47]. BMI was also calculated [48]. Underweight participants (n = 25) were excluded because they were expected to show different trends in the investigated associations than normal weight or overweight participants.

#### Blood pressure

After 10 minutes of rest, blood pressure was measured twice on each arm (OMRON M6 COMFORT, Denmark, bought 2009–2010). If a difference was detected between the four blood pressures (>20 mmHg systolic or >10 mmHg diastolic), a fifth measurement was conducted and average systolic and diastolic blood pressures were calculated from the last two measurements [41].

#### Haemoglobin A1c (HbA_1c_)

A high performance liquid chromatography (HPLC) method was used for determination of HbA_1c_. The HPLC consisted of a Waters 625 LC system together with a Waters photo-diode-array detector model 996 and a WISP 717 auto sampler for automatic injection of the samples. Millennium chromatography software was used for calculation of concentrations (Waters Associates Inc., Milford, United States). A cation exchange column Mono S HR 5/5 from Pharmacia Biotech AB, Uppsala, Sweden was used to separate HbA_1c_ from other components in the samples [49]. HbA_1c_ is a standard indicator of long-term glycaemic control and an HbA_1c_ level of ≥5.6% corresponds to a fasting glucose level of ≥100 mg/dL, which is a measure of insulin resistance [50].

#### HDL and triglycerides

Blood lipids levels were assessed using a COBAS Mira Plus. The measures of HDL and triglycerides were based on ABX Pentra assays from Triolab (Sollentuna, Sweden).

#### Metabolic syndrome score

The metabolic syndrome was characterised by a large waist circumference (≥102 cm for men, ≥88cm for women), dyslipidaemia (triglycerides ≥1.7mmol/L for men or women or HDL <1 mmol/L for men or HDL <1.3 mmol/L for women), a blood pressure of 130/85 mmHg or higher and insulin resistance (HbA1c ≥5.6%, as no fasting blood samples were taken) [12]. For every positive indicator of the metabolic syndrome, one point was added to the overall metabolic syndrome score resulting in a categorical score from 0 to 4.

### Cytokines, C-reactive protein and inflammatory load

Non-fasting blood samples were collected at the day of the clinical examination and stored at -80°C for up to 2 years. An electro-chemiluminescence multiplex system was used to measure the below-mentioned cytokines on a Sector 2400 Imager from Meso Scale Discovery (Gaithersburg, USA) according to the manufacturer’s instructions. IL-6, TNF-α, IL-1β, IFN-γ, and IL-10 were measured in a multi-plex and IL-18 in a single-plex system. Lower limit of detection was the calculated concentration of the signal that was 2.5 standard deviations over the zero calibrator (the blank). All samples were run as duplicates. The intra-assay variation had to be <20% to be accepted. The same two internal laboratory controls were included in all runs: Control A was a fasting plasma sample from a healthy young subject and control B was a plasma sample after endotoxin administration *in vivo*. The same lot number was used for all analyses (IL-18: Z001057; 6-plex: Z000042839), and incubation periods, pipettes, freezing/thawing cycles of buffers and controls were always performed in the same way to limit the inter assay variation as much as possible. Additionally, only two laboratory technicians ran all analyses.

Values below the limit of detection (LOD) for the remaining cytokines were substituted by simple imputation of a value between 0 (zero) and a LOD from a uniform distribution. With regard to TNF-α the in-house LOD was 0.28 pg/ml and the inter assay correlation of variance (CV) in house was 9–13%. For IL-6 the LOD was 0.21 pg/ml and the inter assay CV was 11–21%. Regarding IL-18, LOD was 1.98 pg/ml and inter assay CV was 14–18%. For IL-10 LOD was 0.21pg/ml and inter assay CV was 15–28% and for INF-γ the LOD was 0.19 pg/ml and the inter assay CV was 24–28%.

Levels of hsCRP was assessed with a high sensitive assay (Tina quant, Roche Diagnostics GmbH, Mannheim, Germany) using latex-entrenched immune-turbidimetry analysis (Roche/Hitachi automatic instrument COBAS®), measuring hsCRP.

In total, data from blood samples were missing on 90 participants. In 51 cases, it was not possible to draw blood from the participants. In 39 cases, the participants were excluded after analysis of the blood due to outlying plasma values (hsCRP ≥40 (2 participants), IL-18 ≥775 (15 participants), IL-10 ≥170 (19 participants), IL-6 ≥400 (3 participants)), as these values must be assumed to reflect acute inflammation and expected to show different associations than the rest of the cohort.

#### Inflammatory load

The inflammatory load variable included hsCRP, IL-6, IL-18 and IL-10. The inflammatory load variable was categorical (0–4) and one point was added for every pro-inflammatory biomarker that had a high value (hsCRP≥3mmol/L, IL-6≥75% percentile and IL-18 ≥75% percentile) or if the anti-inflammatory biomarker had a low value (IL-10≤25% percentile). The cut-off limit for hsCRP was chosen to be 3mg/L, since this limit is correlated with high risk of CVD [51]. There is no consensus on relevant cut-off limits for IL-6, IL-18, and IL-10. In the present study, we decided to use quartiles as cut-off limits for these biomarkers. We decided not to include inflammatory biomarkers without a significant effect on a single parameter basis (please see result section) in the inflammatory load variable.

### Covariates obtained from questionnaire

#### Acute inflammatory events

Participants were registered as having experienced acute inflammatory events if they had had broken bones or surgery during the last month, fever, cold, influenza, pneumonia, digestive tract infection or other infections during the last 3 weeks, urinary tract infection during the last 2 weeks or visit to a dentist during the last week prior to the test.

#### Occupational social class

Based on information about occupation, social class was ranked into a categorical variable with 5 groups (I to V) according to the standards of the Danish Occupational Social Class classification [52]. Social class I reflected professional occupation and social class V reflected unskilled occupation. An additional separate category represented people on transfer income, including sickness benefits and disability pension.

#### Smoking and alcohol consumption

Participants were asked to classify themselves as current, previous, or never smokers. The amount of alcohol consumed per week was given in units with 1 unit defined as 1 bottle of beer, 1 glass of wine or 1 drink of strong alcohol.

The entire questionnaire was validated and thoroughly documented. The face validity was established by experts. The questionnaire was pilot tested on a subset of participants prior to the main data collection, and pilot data indicated satisfactory internal consistency. All answers were affirmed.

### Statistics

All statistical analyses were performed using STATA 13.0. T-tests and chi^2^-tests were used to test differences between men and women in the descriptive analyses and between participants and non-participants. Multiple linear regression models were performed to investigate the association between CR-fitness and different measures of abdominal obesity (waist, WHtR, WHR, trunk fat%), adjusted for confounders (age, social class, alcohol consumption, smoking status and sex).

Multiple linear regression models were performed to investigate the association between CR-fitness and different biomarkers of chronic low-grade inflammation (hsCRP, IL-6, IL-18, IL-10, TNF-α, IFN-γ, IL-1β), and the combined inflammatory load, respectively. Analyses were adjusted for confounders (age, social class, alcohol consumption, smoking status, acute inflammatory events and sex). To test if the potential relationship between CR-fitness and inflammation was independent of abdominal obesity, measures of abdominal obesity were added to the model (waist, WHtR, WHR, trunk fat%) one by one.

Multiple linear regression models were performed to investigate the association between CR-fitness and factors of the metabolic syndrome (HbA_1c_, triglycerides, HDL, systolic and diastolic blood pressure) adjusted for confounders (age, social class, alcohol consumption, smoking status and sex). Inflammatory biomarkers were added to the model one by one (hsCRP, IL-6, IL-18, IL-10, TNF-α, IFN-γ, IL-1β and inflammatory load). Interactions between sex and CR-fitness were tested in models with low-grade inflammation or factors of the metabolic syndrome as outcomes to evaluate whether pooling of men and women were justified to obtain maximal power in analyses.

In all regression-models, the assumptions of linearity, homogeneity of variance and normal distributed residuals were tested. Inflammatory biomarkers, factors of the metabolic syndrome and measures of abdominal obesity showed skewed distributions and were consequently log_10_-transformed in regression models. Estimates in regression analyses were back transformed by using the relevant antilogarithm and interpreted as the percentage change in CR-fitness as described by The Institute for Digital Research and Education [53]. We found no other violations of the model assumptions.

## Results

Table 1 provides descriptive characteristics of men and women in the study cohort. The age range was narrow with an age span of only three years due to the inclusion criteria of subjects born between 1959 and 1961. Approximately 38% of the included participants were overweight (BMI≥25) and 15% were obese (BMI≥30). Men had higher levels of CR-fitness, and larger waist, WHR, and WHtR but also higher levels of BMI, IL-18, IL-10, HbA_1_c, triglycerides, systolic and diastolic blood pressure, and metabolic score, as well as lower level of HDL compared to women. The number of current smokers did not differ by sex, whereas previous smokers where more often women.

**Table 1:** Characteristics of the 1,293 participants.

	Men (n = 580)	Women (n = 713)	Total (n = 1,293)	P-value
**Age (years)**	50 (50; 51)	50 (50; 51)	50 (50; 51)	0.3
**CR-fitness (mL/min/kg)**	33.2 (27.6; 40.8)	32.1 (26.9; 37.4)	32.4 (27.3; 38.5)	<0.001
**BMI (kg/m**^**2**^**)**	25.9 (24.0; 28.6)	24.7 (22.2; 27.6)	25.3 (22.9; 28.1)	<0.0001
**BMI≥25, No. (%)**	356 (61)	333 (47)	689 (53)	<0.0001
**Smokers, No. (%)**	Current: 135 (23)Previous: 169 (29)	Current: 160 (23)Previous: 288 (41)	Current: 295 (23)Previous:457 (35)	<0.0001
**Alcohol, No (%)**	>14 units/week: 174 (30)	>7 units/week:246 (35)	>14 or 7 units/week:420 (33)	0.1
**Social class**, N**o (%)**	I: 108 (19) II: 142 (24) III: 143 (25) IV: 82 (14) V: 45 (8) TI*: 43(7)	I: 75 (11) II: 196 (27) III: 160 (23) IV: 138 (19) V: 64 (9) TI*: 61(9)	I: 183 (14) II: 338 (26) III:303 (23) IV:220 (17) V: 109 (8) TI*: 104 (8)	<0.0001
**Acute inflammatory events, No. (%)**	Yes: 190 (33)	Yes: 206 (29)	Yes: 396 (31)	0.1
**Waist (cm)**	96 (90; 104)	87 (80; 95)	91 (84; 100)	<0.0001
**Waist-to-hip ratio**	0.93 (0.90; 0.97)	0.85 (0.80;0.89)	0.89 (0.84;0.94)	<0.0001
**Waist-to-height ratio**	0.53 (0.50; 0.58)	0.52 (0.48;0.57)	0.53 (0.49; 0.57)	0.01
**Truncal fat percentage (%)**	10.4 (8.0; 14.0)	10.2 (7.6; 13.3)	10.3 (7.8; 13.7)	0.1
**HsCRP (mg/l)**	1.1 (0.5; 2.2)	1.0 (0.5; 2.2)	1.0 (0.5; 2.2)	0.3
**IL-6 (pg/ml)**	1.5 (1.0; 2.2)	1.4 (1.0; 2.2)	1.4 (1.0; 2.2)	0.4
**IL-18 (pg/ml)**	289.7(225.2; 364.6)	248.2(194.5; 318.3)	268.3(206.9; 342.9)	<0.0001
**IL-10 (pg/ml)**	1.0 (0.6; 1.8)	0.9 (0.5; 1.6)	0.9 (0.6; 1.7)	<0.05
**TNF-α (pg/ml)**	4.4 (3.7; 5.2)	4.0 (3.4; 4.8)	4.1 (3.5; 5.0)	0.2
**IFN-γ (pg/ml)**	0.4 (0.3; 0.6)	0.4 (0.3; 0.5)	0.4 (0.3; 0.6)	0.7
**IL-1β, No. (%)**	Detectable: 11 (2)	Detectable: 15 (2)	Detectable: 26 (2)	0.8
Inflammatory load**	2 (1; 2)	2 (1; 2)	2 (1; 2)	1.0
**HbA**_**1**_**c (%)**	5.2 (4.9; 5.5)	5.1 (4.8; 5.3)	5.1 (4.9; 5.4)	<0.0001
**Triglyceride (mmol/L)**	1.7 (1.2; 2.5)	1.3 (.9; 1.8)	1.4 (1.0; 2.1)	<0.0001
**HDL (mmol/L)**	52.6 (44.1; 60.7)	63.4 (54.9; 73.9)	58.4 (49.1; 68.8)	<0.0001
**Systolic BP (mmHg)**	132 (123.5; 140)	120.8 (112; 131)	126.5 (116.5; 136.5)	<0.0001
**Diastolic BP (mmHg)**	85.5 (80.5; 92)	83 (76.3; 89)	84.5 (78; 90.5)	<0.0001
**Metabolic syndrome**	3 (2; 3)	2 (2; 3)	2 (2; 3)	<0.0001

Values are presented as median (IQ 25%; IQ 75%) or number (%). *P* values for comparison of sex characteristics are based on independent t-test or chi-square test.

*TI, transfer income.

**Inflammatory load: A combined score of hsCRP, IL-6, IL-18 and IL-10.

### Association between CR-fitness and abdominal obesity

An increase in CR-fitness of +5 mL/min/kg was associated with a decrease in waist (-3.65%; 95%CI: -4.0 to -3.3; p<0.0001), WHtR (-3.65%; 95%CI: -4.0 to -3.3; p<0.0001), WHR (-1.45%; 95%CI: -1.65 to -1.25; p<0.0001) and trunk fat% (-15.1%; 95%CI: -16.4 to -13.85; p<0.0001) after adjustment for age, sex, social class, alcohol consumption and smoking status.

### Association between CR-fitness and biomarkers of inflammation

**Fig 1:**
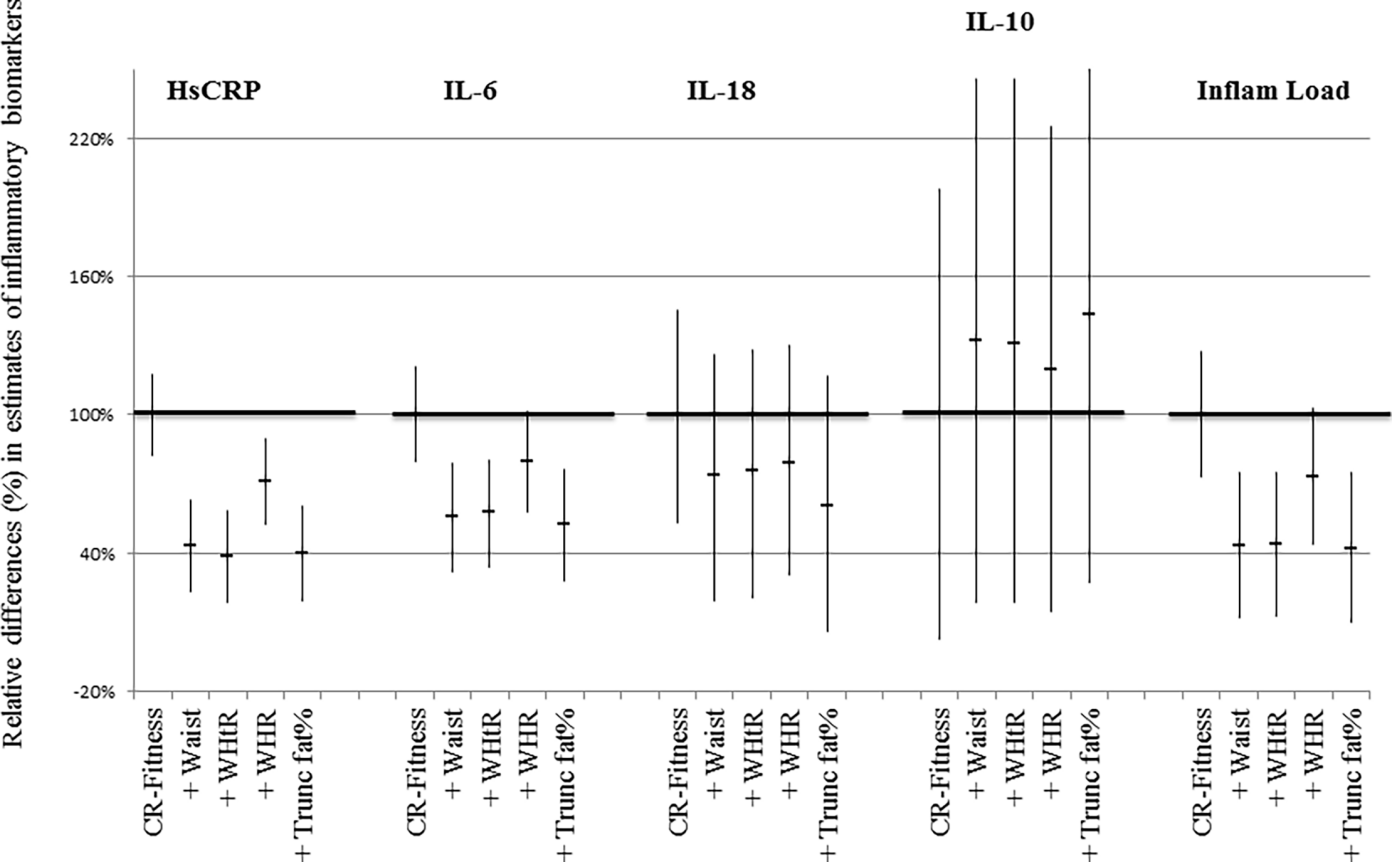
Associations between cardiorespiratory fitness (CR-fitness) and inflammatory biomarkers. Multiple linear regression analyses were performed with CR-fitness as exposure and plasma levels of inflammatory biomarkers as outcomes, adjusted for the effect of age, sex, social class, alcohol consumption, smoking status and acute inflammatory events. Relative differences (%) in estimates of inflammatory biomarkers, adjusted for measures of abdominal obesity. Y-axis: Regression estimates of inflammatory biomarkers from models containing abdominal obesity have been normalised to estimates from the initial model (100%) to show relative percentage changes. P<0.05 on all regression estimates shown. Waist = waist circumference; WHtR = waist-to-height-ratio; WHR = waist-hip-ratio; trunk fat% = truncal fat percentage; MS = metabolic syndrome.

An increase in CR-fitness of +5 mL/min/kg was associated with a decrease in hsCRP (-18.25%; 95%CI: -21.45 to -15.05; p<0.0001), IL-6 (-10.45%; 95%CI: -12.6 to -8.35; p<0.0001), IL-18 (-2.65%; 95%CI: -3.85 to -1.4; p<0.0001), the overall inflammatory load (-5.6%; 95%CI: -7.15 to -4.1; p< 0.0001) and an increase in IL-10 (4.4%; 95%CI: 0.1 to 8.7; p = 0.04), adjusted for the effect of age, sex, social class, alcohol consumption, smoking status and acute inflammatory events (Table 2). There were no associations between CR-fitness and TNF-α, IFN-γ or IL-1β, respectively (data not shown). When different markers of abdominal obesity were added to the model, the estimate for inflammation decreased (Fig 1). Only one statistically significant interaction was found between CR-fitness and sex. This was in relation to IL-6. An increase in CR-fitness of +1 mL/min/kg was associated with a decrease in IL-6 in both men (-1.44%; 95%CI: -2.05 to -0.83; p<0.0001) and women (-2.80%; 95%CI: -3.39 to -2.21; p<0.0001).

**Table 2:** Associations between cardiorespiratory fitness (CR-fitness) and inflammatory biomarkers.

	Total (n = 1,293)		
Model	% difference	95% CI	P-value
**HsCRP**	-3.65	-4.29; -3.01	<0.0001
**IL-6**	-2.09	-2.52; -1.67	<0.0001
**IL-18**	-0.53	-0.77; -0.28	<0.0001
**IL-10**	0.88	0.02; 1.74	<0.05
**Inflammatory load**	-1.12	-1.43; -0.82	<0.0001

Multiple linear regression analyses were performed with CR-fitness as exposure and plasma levels of inflammatory biomarkers as outcomes, adjusted for the effect of age, sex, social class, alcohol consumption, smoking status and acute inflammatory events. Inflammatory load: A combined score of hsCRP, IL-6, IL-18 and IL-10.

### Association between CR-fitness and factors of the metabolic syndrome

**Fig 2:**
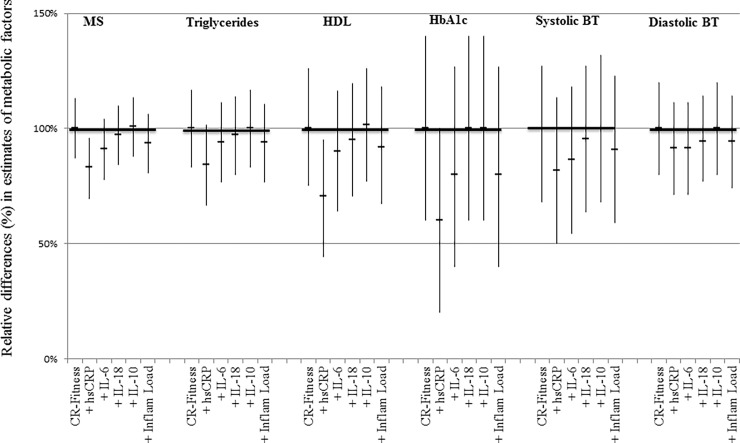
Associations between cardiorespiratory fitness (CR-fitness) and factors of the metabolic syndrome. Multiple linear regression analyses were performed with CR-fitness as exposure and factors of the metabolic syndrome as outcomes, adjusted for the effect of age, sex and social class, alcohol consumption, smoking status. Relative difference (%) in estimates of factors of the metabolic syndrome, adjusted for measures of inflammation Y-axis: Regression estimates of metabolic syndrome factors from models containing inflammation have been normalised to estimates from the initial model (100%) to show relative percentage changes. P<0.01 on all regression estimates shown. Inflammatory load is a combined score of hsCRP, IL-6, IL-18 and IL-10.

An increase in CR-fitness of +5 mL/min/kg was associated with a decrease in the overall metabolic syndrome score (-11.2%; 95% CI: -12.65 to -9.75; p<0.0001), triglycerides (-8.95%; 95% CI: -10.45 to -7.45; p<0.0001), HbA_1_c (-0.75%; 95%CI: -1.05 to -0.45; p<0.0001), systolic blood pressure (-1.1%; 95% CI: -1.4 to -0,75; p<0.0001) diastolic blood pressure (-1.75%; 95% CI: -2.1 to -1.4; p<0.0001) and an increase in HDL (3.05%; 95% CI: 2.25 to 3.85; p<0.0001), adjusted for age, sex, social class, alcohol consumption and smoking status (Table 3). When hsCRP or IL-6 were also added to the models, estimates of metabolic syndrome factors were reduced, indicating that these pro-inflammatory biomarkers could explain part of the association between CR-fitness and factors of the metabolic syndrome. Inclusion of IL-18 and IL-10 had a minor or no effect on the association between CR-fitness and factors of the metabolic syndrome (Fig 2). TNF-α, IFN-γ and IL-1β showed no effect on the associations (data not shown). The overall inflammatory load was mainly driven by hsCRP. No interaction was found between CR-fitness and sex in these regression models.

**Table 3:** Associations between cardiorespiratory fitness (CR-fitness) and factors of the metabolic syndrome.

	Total (n = 1,293)		
Model	% difference	95% CI	P value
**Metabolic syndrome**	-2.24	-2.53; -1.95	<0.0001
**Triglycerides**	-1.79	-2.09; -1.49	<0.0001
**HDL**	0.61	0.46; 0.77	<0.0001
**HbA**_**1**_**c**	-0.15	-0.21; -0.09	<0.0001
**Systolic BP**	-0.22	-0.28; -0.15	<0.0001
**Diastolic BP**	-0.35	-0.42; -0.28	<0.0001

Multiple linear regression analyses were performed with CR-fitness as exposure and an overall metabolic syndrome score or factors of the metabolic syndrome as outcomes, adjusted for the effect of age, sex, social class, alcohol consumption and smoking status.

### Missing and excluded data

Underweight participants (BMI≤18.5, n = 25) had significantly higher levels of hsCRP than the rest of the participants in the CAMB cohort (57.2% difference in hsCRP levels; 95%CI 79.9 to 8.6; p<0.05). Participants with outlying plasma values had more often experienced acute inflammatory events than participants who did not have outlying plasma values (Pearson chi^2^ = 3.9; p <0.05). However, by excluding the outlying plasma values (n = 39), the regression estimates for inflammatory biomarkers only changed by the 3rd and 4th decimal. Participants who did not perform a CR-fitness test had a higher level of hsCRP (51.2% difference in hsCRP levels; 95%CI 26.5 to 80.7; p<0.0001) and a larger waist (4.5% difference in waist; 95%CI 2.3 to 6.8; p<0.0001) than the group who did perform the test. Participants with unsuccessful blood sampling were no different than the rest of the cohort with regard to CR-Fitness level (-4.4% difference in CR-Fitness; 95%CI 4.5 to -14.2; p = 0.3) and acute inflammatory events (Pearson chi^2^ = 0.59; p = 0.4). Participants with data missing on CR-Fitness level because of technical problems with the bikes were not different with regard to inflammation level (hsCRP) (-0.1% difference in hsCRP; 95%CI 20.9 to -26.7; p = 1 and body composition (waist) (0.06% difference in waist; 95%CI 2.9 to -2.9; p = 1) from the rest of participants.

## Discussion

In this cohort study of middle aged Danes from CPC, an inverse association was found between CR-fitness and levels of hsCRP, IL-6, IL-18 and a direct association was found between CR-fitness and levels of IL-10. Abdominal obesity could partly explain these associations and the impact of abdominal obesity was robust and found throughout waist, WHtR, WHR, and trunk fat%. In addition, higher CR-fitness levels were associated with lower measures of abdominal obesity. Moreover, a higher CR-fitness level was associated with a lower overall metabolic syndrome score as well as a beneficial level of all individual factors of the metabolic syndrome (triglycerides, HDL, HbA_1_c, systolic and diastolic blood pressure). Low-grade inflammation could partly explain these associations. These findings support the hypotheses that a high CR-fitness level promotes health by preventing abdominal obesity and lower systemic inflammation which reduces the risk of the metabolic syndrome and thereby the risk of developing diseases such as CVD and T2DM. The impact of inflammation was strongest when hsCRP or IL-6 was used as biomarkers of systemic inflammation.

The association between CR-fitness and hsCRP was in accordance with findings in studies of smaller cohorts [54–56]. The present study contributes with a large cohort, detailed inflammatory data and adjustments for possible confounders, supporting that the anti-inflammatory effect of CR-fitness is partly mediated by a reduction in abdominal obesity. In accordance with data in the present study, human experimental studies using sedentary lifestyle interventions in young healthy males have shown to increase the amount of visceral adipose tissue, decrease the level of fitness, and impair glycaemic control [2,57,58]. Since in our present study the anti-inflammatory effect of a high CR-fitness level was only partly explained by abdominal obesity, we speculate that physical activity *per se* also plays an important role by inducing anti-inflammatory cytokines. Thus, it has previously been demonstrated that each bout of physical activity represents a natural, strong anti-inflammatory response as contracting muscle releases IL-6 which induces a subsequent increase in the production of IL-1 receptor antagonist (IL-1ra) and IL-10, thus stimulating the occurrence of anti-inflammatory cytokines [24]. IL-1ra inhibits IL-1 signal transduction [59], and IL-10 is capable of inhibiting synthesis of pro-inflammatory cytokines such as TNF-α [60]. Moreover, muscle released IL-6 inhibits TNF production [61].

The present study demonstrates that chronic inflammation may in part explain the association between CR-fitness and factors of the metabolic syndrome. In consistence with this finding, associations between inflammation and the metabolic syndrome have previously been documented [62,63] and so has the beneficial impact of CR-fitness on the inflammatory response in patients with metabolic syndrome [64]. The latter study [64] evaluated white blood cell count as biomarker of inflammation whereas the data presented here include an extensive list of inflammatory biomarkers with known biological and/or epidemiological impacts on the metabolic syndrome [65,66]. Measures of hsCRP and/or IL-6 are often used as biomarkers of inflammation in epidemiological studies, although cytokines such as IL-18, IL-10, TNF-α, IFN-γ and IL-1β could be more important from a pathophysiological point of view. However, despite the inclusion of several inflammatory biomarkers and the suggested combination of these into an overall score of the inflammatory load, hsCRP was still the strongest biomarker in relation to CR-fitness and the metabolic syndrome. Still, hsCRP is probably not the biological driver [67,68]. Importantly, we demonstrated systemic inflammation to be one of the explanatory mediators in the association between CR-fitness and the metabolic syndrome. The recently published CANTOS study showed that anti-IL-1β therapy leads to a lower rate of recurrent cardiovascular events [18] and that anti-IL-1β therapy reduces the risk of and mortality from lung cancer in patients with atherosclerosis [69], supporting the concept of persistent inflammation as a pathological driver in disease networks. The chronic inflammation related co-morbidities may contribute to disability and decreased CR-fitness level, inducing accumulation of visceral fat and thereby enhancing inflammation in a positive feedback loop [16]. In this vicious cycle of persistent inflammation, our data support the notion that physical activity is a possible strong physiological brake of disease development due to the anti-inflammatory and anti-obesity effects.

Underweight middle-aged individuals have a higher prevalence of comorbidity [70] and they were expected to show different associations between the investigated parameters than normal- or overweight participants. When comparing them to the rest of the cohort, the 25 underweight participants had higher levels of hsCRP, confirming the difference and accordingly they were excluded from the analyses. Men and women were pooled to obtain maximal power in this study and sex was included in all analysis. Possible interactions between sex and CR-fitness were explored and only one interaction was found significant. This interaction was in relation to IL-6 and since the trend was similar for men and women, it was decided to keep men and women pooled. Considering the large number of tests, this one significant association could be a chance finding, however, that has to be confirmed in future studies.

Limitations of the study must be taken into account. This study represents a narrow age range and only includes participants of BMI>18.5. Thus, our results cannot be generalized to other age groups or underweight individuals. CR-fitness levels were calculated from submaximal tests made on an ergometer bicycle [42], which are not as exact as a VO_2_max test. However, it is well-documented that results from submaximal tests correlate well with results from VO_2_max tests [71]. In addition, as the submaximal test is less challenging, it increases the number of middle-aged individuals who will be able to perform the test successfully. Thirty-one percent of participants had experienced acute inflammatory events in the weeks preceding the data collection (Table 1). This could potentially influence the level of low-grade inflammation. All statistical models containing biomarkers of inflammation were therefore adjusted for this factor. However, if any residual confounding relates to this measurement, this could potentially influence our results and lead to an underestimation of the association between fitness and inflammation. Residual confounding could exist if our measurement of acute inflammatory events did not capture all potential cases of such. If we excluded the 31% participants categorized with an acute inflammatory event and repeated the presented analysis, the trend and significance level of the associations did not change, which indicates that a potential residual confounding is not an issue in this context. The exclusion of participants with missing data on CR-fitness, missing blood samples, or outlying plasma values could potentially cause a selection bias. As expected, the group of participants who were excluded due to the pre-defined criteria from the CR-fitness test had higher levels of hsCRP and a larger waist than the group who did perform the test. Participants with data missing on CR-fitness due to technical problems with the bikes did not have different hsCRP levels or waist than the rest of the participants. Likewise, participants who had unsuccessful blood drawings had no different CR-fitness levels or acute inflammatory events than the rest of the cohort, while participants with outlying plasma values were registered with more acute inflammatory events than the rest of the cohort. These findings support the assumption that outliers in plasma levels of inflammation reflect various acute health conditions rather than chronic low grade inflammation. The bioimpedance analysis also had some limitations as it is influenced by time and hydration status. Bioimpedance has, however, shown to provide reliable estimates of truncal fat mass in middle-aged and older subjects [72]. Finally, the cross-sectional study design does not allow us to draw conclusions on the causal directions of the found associations. In order to conclude on causality, the results of this study need to be confirmed in follow-up or in human intervention studies.

In conclusion, CR-fitness and low-grade inflammation were inversely related and this association was partly explained by abdominal obesity in middle-aged individuals. Moreover, associations between CR-fitness and factors of the metabolic syndrome could in part be explained by systemic low-grade inflammation. These findings support the notion that the beneficial effect of physical activity on metabolic health is partially due to an anti-inflammatory effect of CR-fitness mediated by a reduction in abdominal obesity. A combined score of inflammatory biomarkers was not more robust in mathematical models than single measurements of hsCRP and IL6.
